# From molecules to medicine: the gut‒heart axis comes of age

**DOI:** 10.1080/19490976.2026.2731684

**Published:** 2026-09-19

**Authors:** Tanya Pretorius, Francine Z. Marques

**Affiliations:** a Department of Pharmacology, Cardiovascular Disease Program, Biomedicine Discovery Institute, Faculty of Medicine, Nursing and Health Sciences, Monash University, Clayton, VIC, Australia; b Victorian Heart Institute, Monash University, Clayton, VIC, Australia; c Baker Heart and Diabetes Institute, Melbourne, VIC, Australia

The gut microbiome has a documented role in human health. The emergence of the gut‒heart axis as a concept was a turning point in understanding the mechanisms underpinning cardiovascular health and disease.^1^ While modifiable risk factors for cardiovascular disease, such as smoking, obesity, and poor diet, are well established,^2^ they fail to capture the full spectrum of cardiovascular risk. The gut microbiome has been increasingly recognized as a key driver of this residual risk, alongside other environmental exposures, making it a prime target for cardiovascular research in recent years.^3-6^

The gut–heart axis has entered a new phase, one defined not by correlation but by mechanism, not by association but by molecular causality.^1^ This special collection in *Gut Microbes* assembles commissioned reviews, original research, and invited contributions that together chart four conceptual frontiers shaping the field: (i) the effects of the gut microbiome beyond the gut epithelial barrier, (ii) the non-metabolic chemical contributions to the gut microbiome architecture, (iii) population dynamics and the ability to use multi-omics to resolve heterogeneity in gut microbial research, and (iv) pioneering microbe-based precision interventions. This editorial summarizes the key findings of the gut‒heart axis collection (Figure 1).

**Figure 1.**
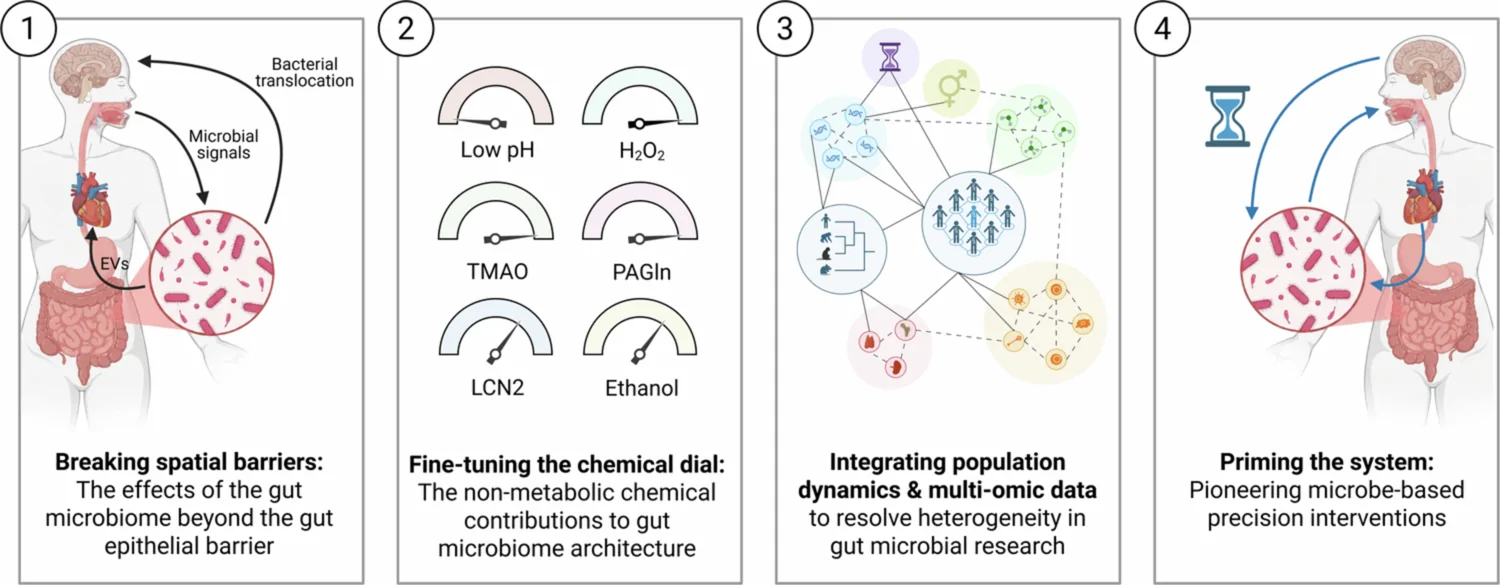
Schematic of the four key topics explored in this special collection: (1) breaking spatial barriers, (2) fine-tuning the chemical dial, (3) population dynamics and multi-omics integration, and (4) precision interventions and priming the system. Created with Biorender.com.

## Theme 1: breaking spatial barriers

Rather than existing in isolation, this theme highlights how the effects of the gut microbiome extend to systemic tissues and organs beyond the gut epithelial barrier. Li et al.^7^ show that an oral pathobiont remodeled gut physiology at a distance: oral *Fusobacterium nucleatum* promotes gut *Lactobacillus* abundance and histidine metabolism, generating imidazole propionate (ImP) that worsens myocardial ischemia/reperfusion injury (MIRI). Mechanistically, they attributed this to impaired cardiomyocyte autophagy, in which imidazole propionate activates p62/mTOR/S6K1 and reduces insulin receptor substrate (IRS) signaling, thereby exacerbating perfusion injury.^7^

This trans-spatial effect is not limited to metabolites—bacteria and their components can themselves cross into the bloodstream and reach systemic tissues. Two primary research papers in this collection identify such mechanisms. Wang et al.^8^ colonized mouse intestines with extracellular vesicles (EVs) from *Escherichia coli* to probe their role in MIRI. EV exposure significantly weakened gut epithelial barrier integrity, allowing EVs to reach cardiac tissue directly, where they acted as carriers of lipopolysaccharide dissemination and triggered inflammation in both the heart and the blood.^8^ The authors also point to glucagon-like peptide-2 as a candidate therapy; by strengthening the intestinal barrier post-MIRI, it limited EV translocation and its downstream inflammatory effects.^8^

Peh et al.^9^ extended this barrier-crossing concept to the brain, providing some of the first evidence that gut bacteria—including core gut commensals *Parabacteroides* and *Barnesiella*—translocate to the brain following stroke, driven by both disrupted gut barrier integrity and pronounced dysbiosis within the first 24 h post-stroke. Using the *β*-blocker propranolol, they implicated the sympathetic nervous system in this process, linking it to worsened grip strength and motor coordination in mice post-stroke.^9^ This mechanistic link suggested a therapeutic angle—propranolol improved motor outcomes in mice by reducing gut permeability and, in turn, limiting bacterial translocation.^9^

## Theme 2: fine-tuning the chemical dial

As evidence for the gut‒heart axis continues to accumulate, attention is shifting from *whether* gut-derived chemical signals affect cardiovascular physiology to *which* specific receptors, metabolites, and microenvironmental cues mediate these effects. The contributions in this theme trace that shift, moving from broad metabolite classes to the precise molecular nodes through which gut chemistry translates into cardiovascular outcomes.

Dinakis et al.^10^ suggest that colonic pH itself is a chemical signal that shapes both microbiome architecture and blood pressure, as evidenced by patients with hypertension, who exhibit a higher maximum colonic pH.^11^ In acidic intestinal environments, produced by acidic microbial metabolites such as short-chain fatty acids (SCFAs), G protein-coupled receptor 65 (GPR65) lowers blood pressure by dampening inflammation via CD8^+^ T-cell activation.^12^ A second receptor, GPR68, is also involved in how low pH induced by SCFAs lowers blood pressure; mechanisms are still being investigated but appear to be immune-independent.^13^ Critically, the authors distinguish this pH-driven pathway from the blood-pressure effects of SCFAs generated by fiber fermentation—both contribute, but through separate mechanisms.^10^ ^,^ ^14^

Torres, Wilcox, and Tang^15^ review trimethylamine *N*-oxide (TMAO)'s role across inflammation, endothelial dysfunction, thrombosis, and arrhythmogenesis. Its causal significance in the gut‒heart axis remains contested, but its reach across many cardiovascular processes makes it a prime target for metabolite-directed therapy. Xu et al.^16^ extended this logic to a wider panel of uremic toxins, including *p*-cresol sulfate/glucuronide, indoxyl sulfate, phenylacetylglutamine (PAGln), and TMAO, which are implicated in cardiovascular-kidney-metabolic syndrome. Their review is notable for tracing how a single class of gut-derived metabolites can drive toxicity across multiple organ systems at once, reinforcing that “fine-tuning” this axis will require targeting shared upstream chemistry rather than organ-specific downstream effects.^16^

Zhang et al.^17^ identified a precise molecular node linking gut chemistry to age-related heart failure. The commensal butyrate producer *Faecalibacterium prausnitzii* mitigates ferroptosis-driven cardiac dysfunction, acting through lipocalin-2 (LCN2)—a butyrate-sensitive regulator of iron accumulation and the oxidative damage that drives ferroptosis.^17^ The authors propose that LCN2 itself is a biomarker, with circulating levels predicting cardiomyocyte senescence and, by extension, cardiovascular risk.^17^ This level of single-node precision is a prime example of the field's shift from broad microbiome associations toward mechanistic, quantifiable chemical signaling.

## Theme 3: population dynamics and multi-omics integration

Established mechanisms underpinning the gut‒heart axis are often generalized across populations, but without accounting for stratification, the field risks reinforcing association rather than isolating true mechanisms. Tang et al.^18^ pointed to one root of this problem: much of the mechanistic evidence base still rests on animal models—rodent, swine, or non-human primate—whose microbiota and physiology do not fully replicate the human system. Even when mechanisms are confirmed in humans, findings from single-population cohorts are obscured by the heterogeneity introduced by geography, migration, culture, and host genetics into the gut microbiome.^18^ The authors argue that multi-ethnic, longitudinal cardiovascular microbiome trials are needed to distinguish between conserved and population-specific mechanisms.^18^

A further layer of complexity is the evolution of gut microbial trajectories over the lifespan, particularly between males and females. Wang et al.^19^ reviewed sex-specific differences in the gut microbiome and metabolome, emphasizing sex steroid hormones as primary drivers. Until menopause, females typically maintain a cardioprotective gut microbial profile; however, the postmenopausal decline in estrogen shifts the microbiome towards a male-like profile, elevating cardiometabolic risk. Ultimately, the authors underscore the necessity of accounting for age, sex, and hormonal status in gut–heart research to move the field beyond its current associative nature.

Gawałko et al.^20^ use atrial fibrillation as a case study to illustrate a broader plateau the gut–heart field faces: associations are accumulating but are not being resolved into consistent mechanisms. Notably, they suggest that metabolites offer more mechanistic traction than taxonomy alone—taxonomic shifts have proven difficult to translate into causal explanations, whereas metabolite pathways at least offer testable, mechanistic hypotheses.^20^ Similarly to Tang et al.^18^ noted that germ-free and animal models remain useful for early-stage discovery but argue that the field’s real bottleneck is the absence of human interventional trials needed to move from genus-level associations to disease-level mechanisms.

Stanford et al.^21^ offer one answer to that call by comparing a “healthy” Australian diet (meeting recommended intakes across the five core food groups) against a typical Australian diet higher in saturated fat, sodium, and sugar and lower in fiber in a randomized clinical trial. The healthy diet enriched microbial biosynthetic pathways consistent with functional reconfiguration of the gut microbiome, and these shifts tracked favorable changes in cardiovascular markers, including plasma cholesterol, triglycerides, and blood pressure, via bile acid, taurine, and diacylglycerol metabolism.^21^

Multi-omics integration in human samples can also help to close the gap between animal studies and translation of findings. Masiá & Gutiérrez^22^ illustrated how combining metagenomics, metabolomics, and proteomics can trace directional pathways from microbial genes to host effector networks, thereby illuminating the roles of TMAO, PAGln, and ImP in atherogenesis. Notably, they position people living with HIV as an essential model population where gut barrier disruption, dysbiosis, and chronic inflammation intersect to accelerate atherosclerotic risk.^22^

Another study by Shi et al.^23^ integrated publicly available faecal metagenomic and host transcriptomic datasets, inferring microbe–metabolite and metabolite–transcriptome relationships in atherosclerosis, moving beyond taxonomic association toward a testable functional model. They identified two convergent, disease-promoting routes: accumulation of H_2_O_2,_ driven by *Bacteroides* depletion, inhibits glutathione peroxidase 2 (GPX2), and depletion of ethanol, driven by enrichment of four other genera, removes its protective activation of Fanconi anemia group D2 protein (FANCD2)—both genes were downregulated in atherosclerosis patients, giving a rare example of a microbe-to-host gene pathway built from observational data.^23^

Together, these studies demonstrate that multi-omics integration delivers unprecedented resolution. By moving beyond high-level associations toward granular, stratified mechanisms, the field will finally account for ethnicity, sex, and host physiology, paving the way for targeted, personalized interventions in cardiovascular health.

## Theme 4: precision interventions and priming the system

As the field matures from mapping mechanisms to acting on them, attention is turning to whether we can actively reshape the gut–heart landscape through nutrition, probiotics, and early development. The contributions in this theme test that premise directly: can priming the microbiota at the right time and with the right “lever” reduce cardiovascular risk before disease takes hold?

Gacasan et al.^24^ show that the *Lactococcus lactis* subsp. *cremoris* (LLC) does more than support gut epithelium integrity—it exerts systemic, cardioprotective effects following ischemia‒reperfusion injury. Their multi-omic analysis revealed that LLC supplementation enhanced glutathione metabolism and fatty acid degradation, reprogramming redox balance and energy homeostasis in the injured myocardium to support recovery.^24^ The authors note that LLC likely acts alongside other microbial metabolites, including the SFCA butyrate, suggesting that targeted probiotic strategies may need to draw on multiple metabolites rather than a single agent.

Lépine et al.^25^ pushed this further, identifying the specific dietary lever responsible. In a randomized clinical trial comparing a plant-protein-enriched diet to an animal-based control, they found the gut microbiota's tryptophan metabolism was redirected away from the tryptophanase pathway and toward the kynurenine pathway, thereby reducing the production of the uremic toxin indole and, with it, inflammation, which is implicated in cardiovascular risk. The authors attributed this shift to the plant-protein diet's higher fermentable fiber content, which also boosts SCFA production associated with cardiometabolic benefits.^25^ Together, these findings position fermentable fiber as a genuine “precision lever”: a single, modifiable dietary input mapped to a defined microbial and metabolic mechanism.

## Conclusion

Taken together, these studies trace real progress in the gut‒heart axis, from crossing anatomical barriers to identifying precise molecular “dials” implicated in cardiovascular health. But the field now sits at an inflection point, as further progress depends less on accumulating associations and more on choosing where to focus next—which populations, which molecular levers, and which windows of biological opportunity matter most. With precision medicine as the end-goal, the studies in this issue suggest three priorities: (i) mechanistic claims must be validated across species and stratified by population heterogeneity, (ii) taxonomic description must give way to the specific metabolites and genes that causally link microbe to host, and (iii) interventions must consider timing, since priming the system requires not just the right input but also a host ready to receive it.

## Data Availability

Data sharing is not applicable to this article, as no new data were created or analyzed in this manuscript.
